# An Integrated Computational Approach to Rationalize the Activity of Non-Zinc-Binding MMP-2 Inhibitors

**DOI:** 10.1371/journal.pone.0047774

**Published:** 2012-11-08

**Authors:** Antonella Di Pizio, Mariangela Agamennone, Massimiliano Aschi

**Affiliations:** 1 Dipartimento di Farmacia, Università “G. d'Annunzio” di Chieti, Via dei Vestini 31, Chieti, Italy; 2 Dipartimento di Scienze Fisiche e Chimiche Università di L'Aquila, Via Vetoio, L'Aquila, Italy; Russian Academy of Sciences, Institute for Biological Instrumentation, Russian Federation

## Abstract

Matrix metalloproteinases are a family of Zn-proteases involved in tissue remodeling and in many pathological conditions. Among them MMP-2 is one of the most relevant target in anticancer therapy. Commonly, MMP inhibitors contain a functional group able to bind the zinc ion and responsible for undesired side effects. The discovery of potent and selective MMP inhibitors not bearing a zinc-binding group is arising for some MMP family members and represents a new opportunity to find selective and non toxic inhibitors.

In this work we attempted to get more insight on the inhibition process of MMP-2 by two non-zinc-binding inhibitors, applying a general protocol that combines several computational tools (docking, Molecular Dynamics and Quantum Chemical calculations), that all together contribute to rationalize experimental inhibition data. Molecular Dynamics studies showed both structural and mechanical-dynamical effects produced by the ligands not disclosed by docking analysis. Thermodynamic Integration provided relative binding free energies consistent with experimentally observed activity data. Quantum Chemical calculations of the tautomeric equilibrium involving the most active ligand completed the picture of the binding process. Our study highlights the crucial role of the specificity loop and suggests that enthalpic effect predominates over the entropic one.

## Introduction

Matrix metalloproteinases (MMPs) are a family of 23 zinc- and calcium-dependent endopeptidases in humans, involved in many processes spanning from connective tissue turnover to cellular signalling [1] in both normal and pathological conditions such as cancer, chronic inflammations, atherosclerosis [2]. Among them, MMP-2 (gelatinase A) is considered a relevant target in anticancer therapy because its involvement has been demonstrated in different human tumors [3]. In particular, it plays a key role in angiogenesis and metastasis by degrading type IV collagen, the principal component of basement membranes, and denatured collagen (gelatin) [4], [5], [6], [7], [8].

**Figure 1:**
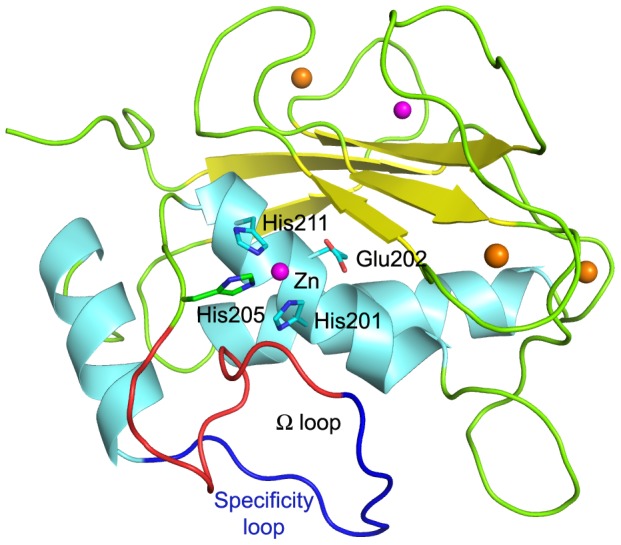
Catalytic domain of MMP-2. The catalytic domain of MMP-2 is formed by five β-strands (yellow), two long α-helices (cyan), unstructured regions (green), and by zinc (magenta) and calcium (orange) ions. Residues of the conserved zinc-binding motif are represented as sticks. The specificity loop, which consists of the residues Tyr223-Gln234 of the Ω-loop (red), is shown in blue.

MMP-2 is a multidomain enzyme made up of a prodomain, a catalytic domain, with an insert of three fibronectin type II repeats, and a hemopexin-like domain. The active site, located in the catalytic domain, contains a conserved zinc-binding motif (HExxHxxGxxH) common to all metzincins and responsible for the coordination of the catalytic zinc ion [7], [8], [9] by three histidine residues (His201, His205 and His211), while the conserved glutamate residue (Glu202) plays an essential role for the catalytic activity [10], [11] (Figure 1).

Because of their role in many pathological conditions, several MMP inhibitors (MMPIs) have been developed but with no success, as their clinical administration caused severe tendonitis-like joint pain, termed “musculo-skeletal syndrome” [12], [13], [14], [15]; this toxicity most probably results from a non specific inhibition of other metallo-enzymes [16], [17].

MMPIs typically comprise a zinc-binding group (ZBG), which binds the catalytic zinc ion, and a moiety that accommodates within the hydrophobic S1′ site. The presence of the ZBG ensures great potency to these inhibitors, but it is responsible for their lack of selectivity and probably for their already mentioned side effects. Consequently, research has been focused on designing selective compounds able to discriminate between different members of the MMP family, exploiting the interaction with the “specificity loop”, the loop surrounding the S1′ site with the highest sequence variability among various MMPs (Figure 1) [17], [18], [19].

**Figure 2:**
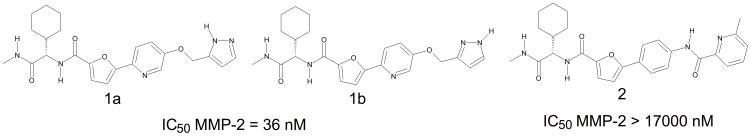
Structures and IC_50_ values of the studied ligands 1a, 1b and 2.

In the last years, a new generation of MMPIs was identified, classified as non-zinc-binding inhibitors. These ligands occupy the S1′ active site deeply and interact with the residues of the specificity loop; therefore they present high selectivity and potency even if they do not bind the catalytic zinc. To date, MMP-8, -12, and -13 selective inhibitors were identified and characterized by crystal structures [20], [21], [22], [23], [24], [25]. Studies carried out on some non-zinc-binding MMP-13 inhibitors demonstrated that, acting by a non-competitive mechanism, they do not induce musculo-skeletal syndrome [20]. Heim-Riether et al. have recently identified non-zinc-binding MMPIs that occupy not only the S1′ but also the S3′ pocket [26]. Although they are quite selective toward the MMP-13, some of them show an interesting activity against the MMP-2, even lacking a zinc-binding group. Because of the relevant therapeutic potential of selective MMP-2 inhibitors, these results prompted us to explore their binding mode on this target because no data on non-zinc-chelating inhibitors of MMP-2 have been disclosed before. In this work, we examined the binding of two ligands, whose MMP-2 activity was determined experimentally [26]. We chose an active ligand, considered in its two tautomeric forms (**1a** and **1b**) during our studies, and an inactive one (**2**), all shown in Figure 2.

In this work we attempted to rationalize the different affinity of **1a**/**1b** and **2** toward MMP-2 using computational approaches somewhat complementary.

Beyond the well documented limitations of the involved force-fields, mechanical-dynamical (thermal) effects are crucial for a physically coherent picture. In fact the receptor flexibility, as well as the solvent effect, strongly affects the study of small-big molecule binding. Moreover proteins in solution exist in a manifold of different conformations and the ligand-protein interaction may cause unpredictable conformational rearrangements [27], [28], [29]. In this respect dynamical approaches are mandatory. As a matter of fact, although many different docking-based approaches have been applied to handle moving targets (e.g. ensemble docking, induced fit methods) and docking software are evolving to account for flexibility [30], the combined use of docking and molecular dynamics (MD) simulations is the most widely used method of investigation [31]. In the present case, the necessity of such a combination of computational tools is even reinforced by the fact that our target macromolecule belongs to a family of flexible enzymes [32], [33], [34], as demonstrated by crystallographic data of MMPs. Moreover they undergo conformational changes upon inhibitor binding [24], [35] as revealed by previous MD investigations [36], [37].

Our study has been initiated performing docking calculations for providing reliable initial structures to be used for subsequent MD simulations which incorporate the flexibility of both the ligand and the receptor, and the solvent [38]. Moreover, MD simulations of free ligands in aqueous solution were compared with those of the inhibitor interacting with the active site, to analyze the effect of enzyme-ligand interaction on ligand fluctuation.

Computational investigation on the thermodynamics of inhibitor binding is not a simple problem with straightforward receipts. In this study the relative binding free energies have been evaluated through Thermodynamic Integration (TI) and compared to the available experimental data for underpinning our analyses and also for identifying plausible dynamical and structural factors determining the activity of both inhibitors.

The different stability of the tautomers **1a** and **1b** and, hence, their actual role in the binding to MMP-2 has been finally studied using Quantum Mechanics (QM) calculations.

## Materials and Methods

Docking and MD calculations were performed on a Fujitsu Siemens Celsius R550 workstation, equipped with two Intel Quad-Core Xeon E5410 2.33 GHz processors.

### Docking

Ligands were manually built in Maestro [39], exploiting the Built facility. The tautomers for the given input structures were produced by the Tautomerizer tool available in Maestro. The protonation state of the ligands were calculated using the Calculator Plugin of Marvin [40].

Ligands were minimized to a derivative convergence of 0.001 kJ/(mol Å) using the Truncated Newton Conjugate Gradient (TNCG) minimization algorithm, the OPLS2005 force field and the GB/SA water solvation model implemented in MacroModel v 9.9 [39]. Conformational searches applying the Mixed torsional/Low-mode sampling and the automatic setup protocol were carried out on all minimized ligand structures in order to obtain the global minimum geometry of each molecule, as the docking program Glide v 5.7 [39], [41], [42], [43] has demonstrated better performances using the global minimum conformation as the ligand starting geometry.

The atomic coordinates of MMP-2 (PDB ID: 1QIB) [44], used in the docking experiments, were recovered from the Protein Data Bank (http://www.pdb.org) [45]. The protein was submitted to the Protein Preparation routine in Maestro that allows to fix the receptor structure by eliminating crystallographic water molecules, adding hydrogen atoms and minimizing the macromolecule structure to optimize the position of hydrogen atoms and eliminate strains. A water molecule was added on the catalytic zinc ion as the fourth binder.

Ligands were docked into the active site of MMP-2 using two accuracy levels available in Glide, the Standard Precision (SP) and the Extra Precision (XP) docking calculations. The most highly ranked poses from the SP mode were submitted to the XP level docking.

### Molecular Dynamics

All the MD simulations were performed using the GROMACS software [46].

The top scored pose for each docked ligand was used as a starting model for MD simulations of complexes, while the MMP-2 structure (PDB ID: 1QIB) was used for MD simulation of the apo form.

In order to eliminate bad contacts in the initial geometry, the obtained complexes and the free enzyme were energy-minimized for 1000 steps using the steepest descent procedure.

The relaxed structures were embedded in a periodic box that extended 15 Å from the protein atoms. The box was then filled with 16210 SPC water molecules [47] to reproduce the typical liquid density. The electroneutrality of the systems was ensured by adding two Na^+^ counterions. Full systems were energy-minimized for 1000 steps of steepest descent minimization, followed by 2000 steps of conjugate gradient minimization until a convergence value of 0.001 kcal/(mol Å) and were slowly heated from 0 to 300 K using short simulations of 500 ps. Each simulation was finally carried out in an isothermal/isochoric ensemble (NVT ensemble) at 300 K for 16 ns using an integration step of 1.0 fs. Coordinates were saved for analysis every 1 ps. For each simulation, the first part of trajectory (1.5 ns) was discarded and only the last 14.5 ns of each trajectory were analyzed.

The temperature (300 K) of the systems was controlled during the MD simulations by Berendsen method [48] used as an isothermal bath, i.e. with the time-constant equal to the integration step.

The zinc atoms were used in the bonded representation [49], the catalytic zinc was covalently linked to His201, His205, and His211 residues plus a water molecule while the structural zinc was covalently linked to Asp153, His151, His166, and His179. For the calcium ions, we used the non-bonded representation proposed by Aqvist [50]. All bond lengths were constrained using the LINCS algorithm [51].

Long range electrostatics were computed by the Particle-Mesh-Ewald (PME) method [52]. Gromos 96 force field parameters [53] were adopted for the receptor, while the Lennard-Jones parameters of similar atoms were considered for the ligands. Essential Dynamics (ED) analysis [54] was carried out to better characterize mechanical-dynamical features of the investigates systems. This method consists of building the covariance matrix of the atomic positional fluctuations to recognize relevant collective (essential) motions. Upon diagonalization of the covariance matrix, a set of eigenvalues and eigenvectors is generated defining a new range of coordinates. The eigenvectors correspond to directions in a 3 N dimensional space (where N is the number of utilized atoms, e.g. typically C^α^ atoms for peptide systems) along which the fluctuation of atoms occurs. The eigenvalues correspond to the mean square fluctuation of the system along the above eigenvectors. Simulations of free ligands in aqueous solution were also carried out adopting the same protocol.

In all the above simulations all the reported data are affected by an uncertainty estimated by means of the standard error calculated utilizing three subportions of the whole trajectory. TI calculations were carried out at two different temperatures, i.e. 300 K and 323 K. As starting configurations we used three different solvated enzyme-ligand structures extracted from ED analysis. We utilized the TI procedure as implemented in Gromacs software, with soft core potential with α = 1.51, σ = 0.3 nm and Δλ = 0.001. The protocol recently proposed by other investigators [55] was adopted for the evaluation of the related errors.

Analysis of H-bond occurrence was carried out using the criterion suggested in the default of the Gromacs package.

### Quantum Chemical calculations

Quantum Chemical calculations were performed using Density Functional Theory applying the hybrid Becke3LYP functional [56], [57] in conjunction with 6–31+G(d) basis set. All the structures were optimized in the gas phase (ideal gas condition) and characterized as true minima or higher order saddle points diagonalizing the mass-weighted Hessian matrix. The same procedure was then utilized to calculate the molecular partition functions for free energy evaluation. Hydration free energies, to be used for reaction free energies in solution, were evaluated in the framework of mean-field approximation in the Conductor-like Polarizable Continuum Model (CPCM) [58], [59]. The calculations were carried out with Gaussian03 package [60].

## Results and Discussion

### Outline of the Computational strategy

In the present work a combined computational approach has been applied in order to clarify the effects produced, on the target macromolecule, by the binding of two structurally related ligands with different affinity toward MMP-2 as well as the structural and dynamical differences of the selected ligands.

The ultimate goal of the computational approach illustrated in this study is to estimate the differential binding free energy of aqueous **1a**, **1b** and **2** toward MMP-2, and its comparison with available experimental data. The calculation of absolute binding free energy is a very complicated task [61] and TI, in this respect, is a widely employed approach [38], [55], [62] whose limitations mainly reside in its high sensitivity to the exhaustive sampling of configurational space (CS) hence requiring advanced computational strategies [63], [64] or, when possible, exhaustive or complete sampling of the CS.

The described protocol, comprising the following steps, has been setup in order to reduce the systematic error associated to a poor sampling of the CS.

– Docking calculations of the studied ligands in the MMP-2 active site were carried out to obtain putative binding poses, that were evaluated and analyzed.
– MD simulations of the free enzyme, the three complexes and the free ligands were carried out and examined.
– The obtained trajectories were subsequently analyzed by means of Essential Dynamics (ED) in order to extract the significant starting configurations to be used for free energy calculations.
– Free energies calculations, initiated by the ED-based basins, were accomplished using TI method at 300 K.
– TI calculations were also performed at higher temperature (323 K) in order to qualitatively estimate the role of enthalpic and entropic factors.
– QM calculations of the stability of the two tautomers **1a** and **1b** were finally carried out in order to definitely assess their occurrence in aqueous solution.

### Docking Calculations

**Figure 3:**
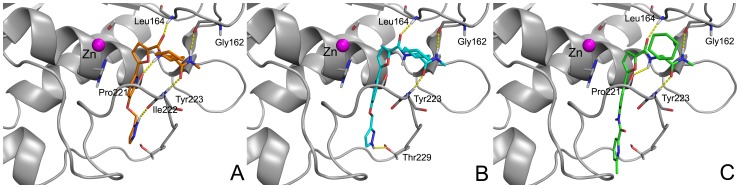
Best docking poses in the MMP-2 binding site of 1a (A), 1b (B) and 2 (C). MMP-2 is represented as solid ribbon; the zinc ion as a purple sphere. H-bond interactions are depicted as yellow dashed lines.

The binding mode of compounds **1a**, **1b** and **2** into the MMP-2 catalytic domain was studied at first through docking calculations. The results show that all ligands, predicted as uncharged at neutral pH, bind MMP-2 adopting a conformation very similar to that experimentally observed for MMP-13 (PDB IDs: 3I7G and 3I7I), spanning from the S1′ to the S3′ sites and not coordinating the zinc ion [26]. On the basis of these data, this would be the first example of non-zinc-binding MMP-2 inhibitors. However, as the presence of the water molecule on the zinc would preclude the binding of the inhibitor to the catalytic zinc, to evaluate the ability of these compounds to coordinate this ion, docking runs were carried out also without the water molecule as the fourth zinc ligand. Poses showing the inhibitors binding the zinc, have much lower docking scores and an unfavourable positioning in the S1′ site, which is widely recognized as the most relevant interaction of MMPIs (data not shown). These results, along with the previously described similarity to the experimental binding conformation in MMP-13, were considered as a validation of docking poses shown in Figure 3 and hereinafter described.

The docking score assigned to the active ligands is higher (−10.0 and −9.23 for ligand **1a** and ligand **1b**, respectively) in comparison with the inactive one (−9.07).

The active ligand establishes with the protein three hydrogen bonds, observed for both tautomers **1a** and **1b**, between: the methyl amide NH and the backbone Gly162 CO, the cyclohexyl amide CO and the Tyr223 NH, the furanyl amide CO and the Leu164 NH. The pyrazyl NH forms a H-bond with the Ile222 CO for the tautomer **1a** and with Thr229 OH for the tautomer **1b**. **1a** establishes an additional H-bond between the furanyl amide NH and the Pro221 CO. The principal hydrophobic interaction in the S1′ site consists of the π-π face to face stacking between the ligand pyridine and the His201 imidazole.

The ligand **2** finds similar H-bonds between: the ligand methyl amide NH and the Gly162 CO, the cyclohexyl amide CO and the Tyr223 NH, the furanyl amide NH and the Pro221 CO, the furanyl amide CO and the Leu164 NH. Moreover the π- π stacking between the ligand phenyl and the His201 in the S1′ subsite can be observed also for this ligand, that does not form any H-bond with the heterocycle ring at the bottom of the S1′ site.

It is clear that docking calculations do not provide an exhaustive rationalization of the different IC_50_ observed for studied inhibitors. In fact, a very similar binding mode for all ligands was obtained, with a comparable docking score. On the basis of these calculations ligand **1a** results a slightly better binder of the enzyme, because it can establish more interactions and shows higher docking score with respect to the others. However these results do not help to explain the lack of activity of ligand **2**, which binds the enzyme with similar interactions.

### MD simulations

#### Dynamical-mechanical and structural features of the whole enzyme upon inhibitors binding

We initially focus our attention on the whole structural and mechanical-dynamical effects produced onto the MMP-2 by the presence of the inhibitors. For this purpose, MD simulations were performed for the free enzyme, the complexes starting from the docked poses, and the free inhibitors.

Note that in the rest of this study the complexes of MMP-2 with the investigated inhibitors will be termed as MMP-2:**1a**, MMP-2:**1b** and MMP-2:**2**.

The stability of the simulations and the qualitative structural behavior of the protein were investigated by calculating the root-mean-square deviation (RMSD) of the C^α^ atoms for all models with respect to the corresponding starting structures. The results indicate that, within the simulated time, the obtained trajectories are rather stable. As a matter of fact, the C^α^ RMSD systematically increases during the simulation and, approximately after 1500 ps, it reaches a plateau at about 0.20 nm for all the systems. The RMSD, when computed for all atoms of the ligands, shows that in all of the cases the ligand roto-translational deviation does not exceed 0.2 nm from the docking pose.

Another important aspect which deserves a careful inspection, is the analysis of the alteration of the internal enzyme framework mobility induced by the presence of the inhibitor. Conformational energy variations and the way in which the fluctuation is distributed within the internal modes have been recognized to be of crucial importance for ligand binding [65]. At this purpose we used two standard and complementary analyses: the root-mean-square fluctuation (RMSF) of the enzyme C^α^ atoms and the ED on the same subset of atoms.

**Figure 4:**
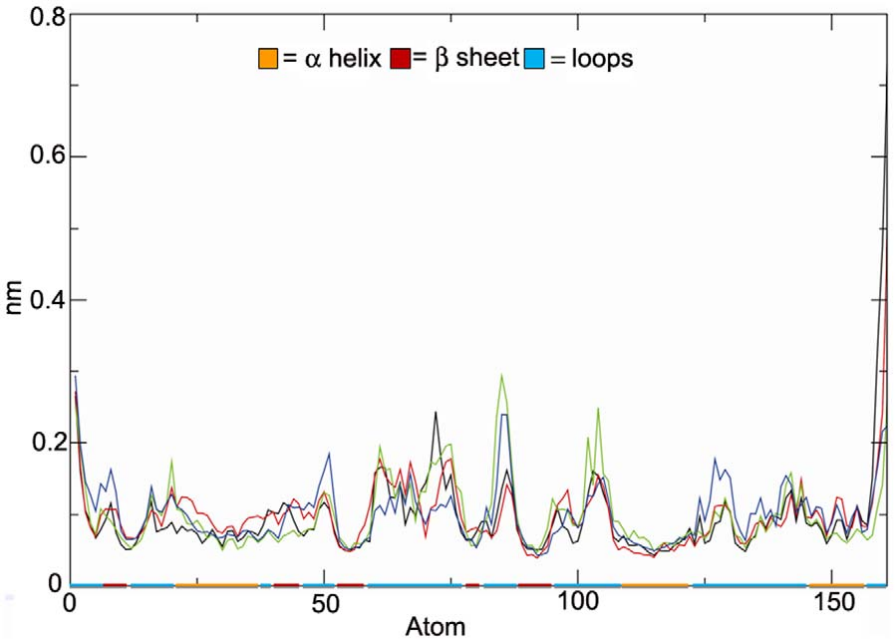
Root-mean-square fluctuations (RMSF, nm) of the MMP-2 C^α^ atoms. RMSF is reported for the free enzyme (blue curve), the complex with **1a** (black curve), with **1b** (red curve) and **2** (green curve). The baseline indicates β-strands (red line), α-helix (orange line) and loops (cyan line).

All the simulations show a comparable RMSF (Figure 4) with largest fluctuations occurring in the unstructured regions, with α-helices and β-strands remaining essentially unaltered. Consistently with this observation, it is also important to note that the secondary structural elements, analysed by the DSSP methods [66] and whose results are not reported for the sake of brevity, are maintained during all simulations.

As shown in Figure 4 the binding of the ligands to the MMP-2 active site does not induce dramatic changes in the global enzyme fluctuation. However some localized modifications in the RMSF pattern might be worth of further attention. A dramatic reduction of fluctuation (i.e. increase of mechanical stability) was found for the S1′ site (see residues 131–144 in Figure 4) in the case of all complexes, and for the S3′ site (see residues 74–76 in Figure 4) for MMP-2:**1a** and MMP-2:**1b**.

**Figure 5:**
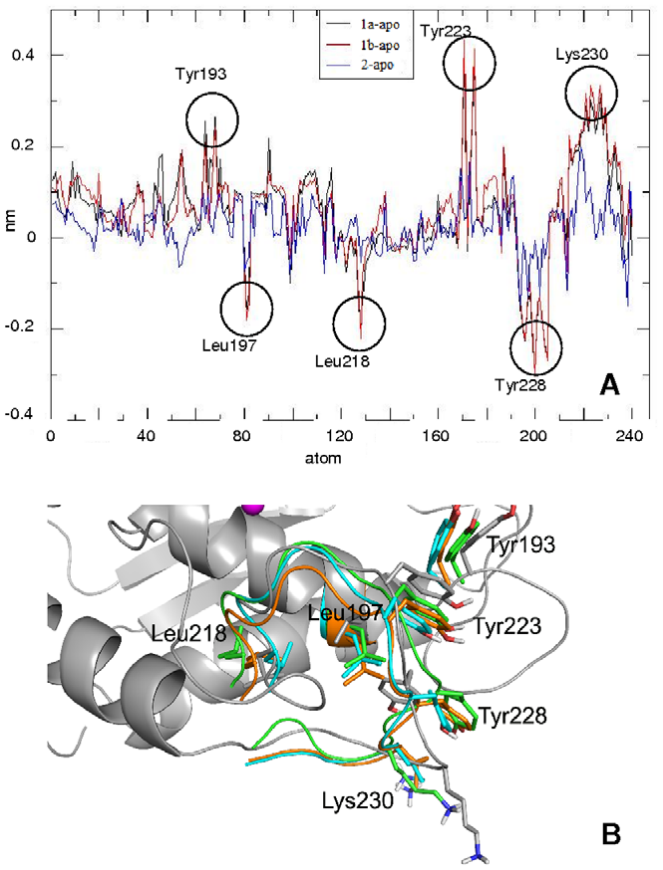
Perturbation on the enzyme structure. A) ΔRMSD for the three simulated complexes. ΔRMSD stands for RMSD_atom-i-complex_-RMSD_atom-i-apo_. The atoms of the S1′ pocket are numbered from 1 to 240; B) Schematic representation of the S1′ pocket. Described residues are represented as sticks.

To get more insight on this aspect we investigated the effect on the average enzyme structure upon binding. This has been accomplished by comparing the single-residue (single C^α^) RMSD of each complex with the corresponding RMSD of the free enzyme. An ideal value of 0.0, hereafter termed as ΔRMSD, implies that the presence of the inhibitor does not significantly alter the structure of the enzyme. The result reported in Figure 5A shows that the lowest ΔRMSD is found for MMP-2:**2**, indicating that the insertion of this inhibitor produces the lowest structural perturbation on the enzyme structure. On the other hand MMP-2:**1a** and MMP-2:**1b**, showing a very similar ΔRMSD, undergo sharp variations in correspondence to the residues Tyr223, Tyr228 e Lys230 all belonging to the S1′specificity loop. Interestingly, occurrence of intra-molecular H-bonds for residues belonging to the S1′ site has revealed that in MMP-2:**1a** (17±1 number of contacts) and MMP-2:**1b** (16+1 number of contacts) this number is larger than the one found for the apo (15±1 number of contacts) and MMP-2:**2** (13±1).

This result implies that the inhibitor **1**, in both the tautomeric forms, produces structural modifications essentially concentrated in the S1′ site, enhancing the number of H-bonds. This stabilization could be taken into account as determining the observed differences in the enzyme-inhibitor affinity.

**Figure 6:**
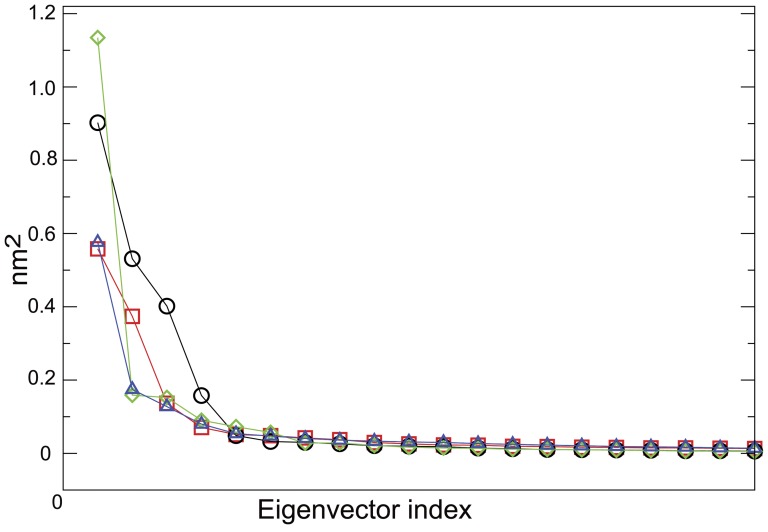
Eigenvalues (nm^2^) of the covariance matrix of MMP-2 C^α^ positional fluctuations. Eigenvalues are reported for the free enzyme (blue triangles) and after complexation with **1a** (black circles), **1b** (red square) and **2** (green diamonds). Estimated standard error, using three subportion of the trajectories, does not exceed the 10% of the reported values. X-axis contains the eigenvector index. Note that only the first 20 eigenvalues are shown.

Further information was derived from ED analysis [54]. Figure 6 reports a significant portion of the covariance matrix spectrum of eigenvalues from the diagonalization of the covariance matrix for the apo and complexed MMP-2.

**Figure 7:**
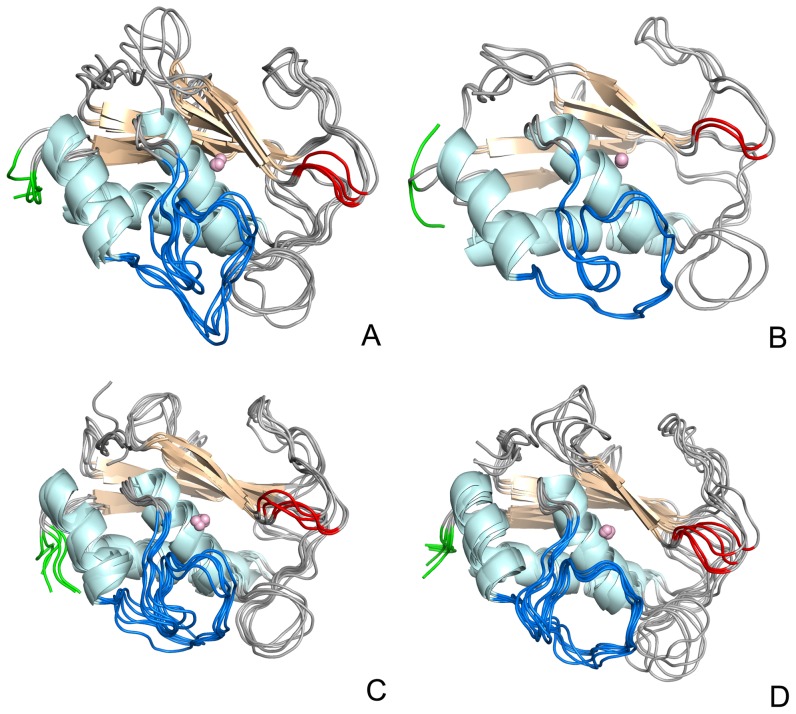
Most sampled C^α^ configurations obtained from ED analysis. A) Free enzyme, B) MMP-2:**1a**, C) MMP-2:**1b**, D) MMP-2:**2**. The Ω-loop is represented in blue, the S3′ loop in red and the C-terminal loop in green.

The trace of the covariance matrix, which quantifies the extent of the whole enzyme fluctuation, turned out to be rather similar (within the error) in all of the investigated cases (2.4±0.2 nm^2^, 2.0±0.2 nm^2^ and 2.0±0.2 nm^2^ for MMP-2:**1a**, MMP-2:**1b** and MMP-2:**2** respectively and 1.9±0.2 nm^2^ for the apo MMP-2). However different steepness emerged in the eigenvalues spectrum (Figure 6). In particular, in the case of MMP-2:**2** only the first eigenvector turns out to significantly contribute to the whole fluctuation. On the other hand, in the apo, MMP-2:**1a** and MMP-2:**1b** also the second eigenvector shows not negligible eigenvalues. The above findings imply that the presence of the ligand, although not producing relevant variations in the extent of the whole enzyme internal fluctuation, alters the repertoire of enzyme conformational space which can be visualized by considering the structures extracted by ED analysis on the whole enzyme (see Figure S1 for details) reported in Figure 7.

Major differences emerge in particular in the unstructured regions (grey loops), in the C-terminal coil (green) which is particularly mobile in MMP-2:**1a**, in the Ω-loop (blue) and S3′ loop (red), whose conformational repertoire appears rather different for the four systems.

Moreover, comparing the diverse conformations of complexed enzymes with the apo form, greater differences concern the S1′ loop. In the apo form, the S1′ pocket adopts a closed state, while in the complexed forms an open state, differently from what described in previous articles [67], [68].

This conformational change is in agreement with the above results and might depend on the ligand dimensions: the S1′ site assumes a tunnel-like shape in the complexes MMP-2:**1a** and MMP-2:**1b**, while it enlarges in the MMP-2:**2**. This behaviour of the MMP-2 binding site can represent an example of induced-fit effect, where the apo form of the protein is not able to explore conformations that can be sampled by the ligand, as expected on the basis of the conformational selection theory [69].

In conclusion the analyses of the enzyme fluctuation confirm the role of the S1′ site motion in the interaction with non-zinc-binding inhibitors [20].

The next step concerns the use of the same computational strategy for determining whether the inclusion into MMP-2 induces, at a similar extent, the same changes into the inhibitor.

#### Dynamical-mechanical features of the free and bound inhibitors

**Figure 8:**
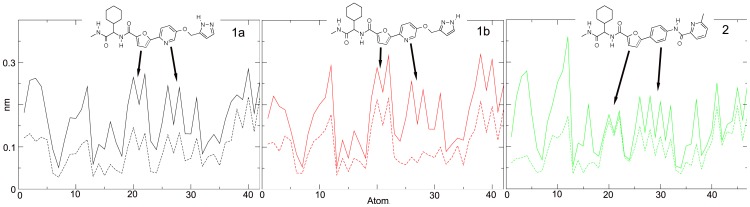
All-atoms RMSF of 1a (black), 1b (red) and 2 (green) in aqueous solution (full line) and bound to MMP-2 (dashed line). Estimated error does not exceed 10% in all the systems.

In Figure 8 and in Table 1 we compared the all-atom RMSF and the trace of the all-atoms covariance matrix of the free and bound inhibitors. In general, the interaction with the enzyme systematically lowers the fluctuation of the inhibitors.

**Table 1:** Sum of eigenvalues (trace) of all-atoms covariance matrix for inhibitors in MMP-2 and in aqueous solution.

ligand	Trace in complex (nm^2^)	Trace in aqueous solution (nm^2^)
**1a**	0.47±0.05	1.4±0.1
**1b**	0.52±0.05	1.3±0.1
**2**	0.51±0.05	2.0±0.2

However, slight but significant differences between the ligand **1a**, **1b** and **2** are worthy of remark. From Table 1 the species **2**, upon insertion into the active site, undergoes the largest loss of internal mobility with respect to the free situation, particularly concentrated in the terminal region characterized by atoms 1–20.

On the other hand, the loss of internal mobility in the case of inhibitors **1a** and **1b** also involves furan and pyridine rings (indicated with arrows in Figure 8).

The local interactions experienced by the three ligands into MMP-2 have been then further analyzed. Evaluation of the average number of H-bonds occurring between inhibitor and receptor, produced indistinguishable values for MMP-2:**1a** (2 H-bonds), MMP-2:**1b** (1 H-bond) and MMP-2:**2** (2 H-bonds). These data do not agree with those obtained from docking calculation, where several H-bonds were established between the ligands and the enzyme. This is not surprising as in the MD system thermal effects as well as the presence of the solvent might severely alter the static picture provided by docking calculations.

**Figure 9:**
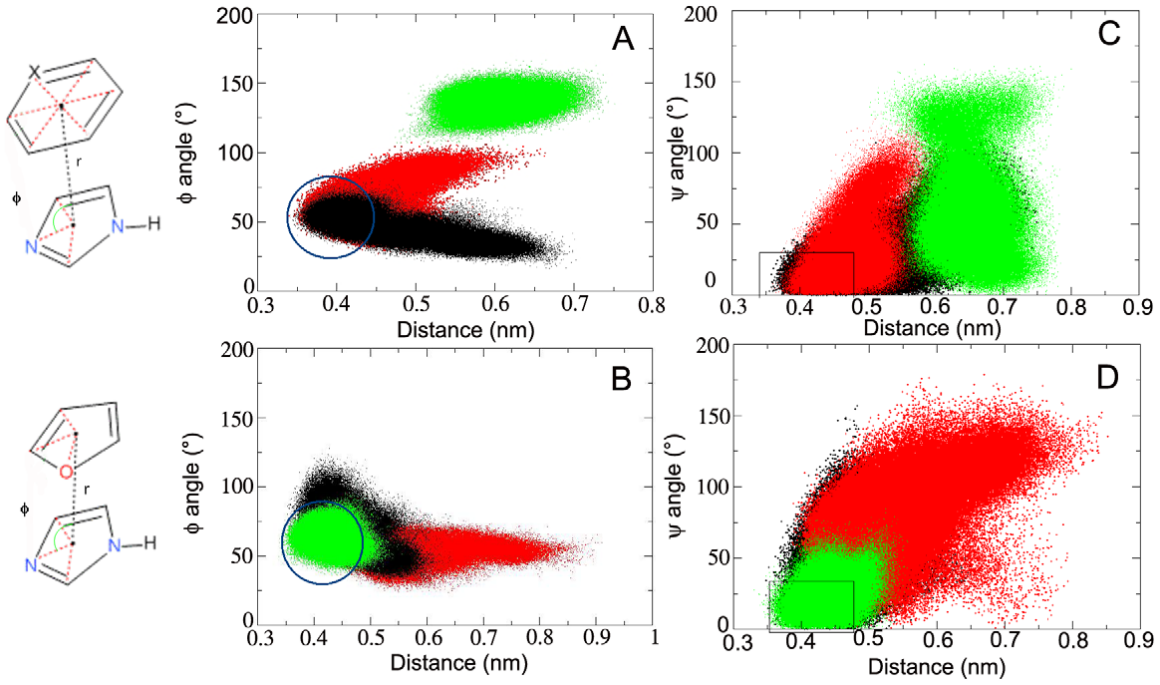
Projection of the trajectory onto φ and r (A and B) and Ψ and r (C and D). Evaluation of the shifting between the His201 and: A) the interacting aromatic moieties; B) the furan ring. Note that ligand **1a** is reported in black, the ligand **1b** in red, and ligand **2** in green. The region in which it is plausible to consider the interaction as formed was highlighted with a blue circle.

On the other hand major differences emerged by analysing inter-aromatic interactions which are presumed to play a crucial role, especially in this case, where the binding site is a hydrophobic pocket. In particular, for non-zinc-binding MMPIs, it has been demonstrated the importance of the π-π stacking interaction with one of the His residues present in the conserved zinc-binding motif, to achieve binding potency [70]. The aromatic groups of ligands, which are able to give the π-π stacking with the His201 of the enzyme, are the pyridine and the furan for the active ligands and the phenyl ring and the furan for the ligand **2**. The interaction of His201 imidazole with these aromatic rings was analyzed measuring the distance between the centre of mass, the shifting and the parallelism between the rings involved in the interaction. The shifting was checked by monitoring the φ angle defined as the unit vector connecting the centre of the mass of the two ring projected onto the imidazole aromatic plane (Figure 9). In our definition the situation in which the two rings face corresponds to a value of φ equal to 90°. The parallelism was checked by considering the Ψ angle between the unit vectors orthogonal to the plane (perfect parallelism with Ψ = 0.0).

The results shown in Figure 9 clarify the different RMSF profiles observed for the three compounds and displayed in Figure 8. In fact, the pyridine of the ligand **1b** results involved in π-π interaction with His201 and its fluctuation decreases when enters the active site. On the other hand, the furan moiety, not interacting with the His imidazole, turns out to be more free to move. For ligand **1a** both pyridine and furan share the π-π interaction with the protein, and their mobility is affected accordingly, while ligand **2** is able to form a close interaction only with the furan ring (Figure 9).

**Figure 10:**
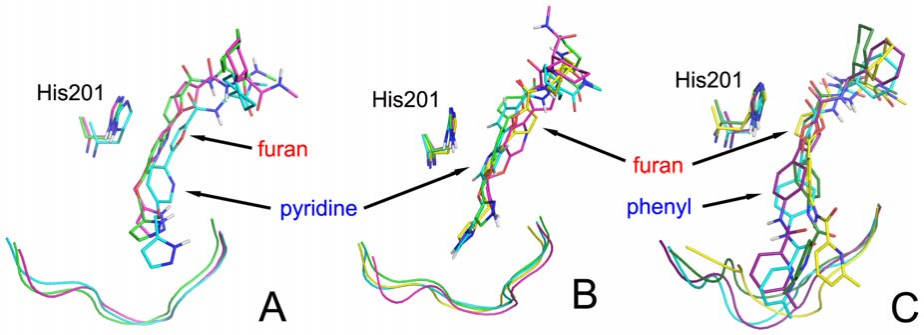
Representative structures for inhibitors, His201 and S1′ site as obtained from ED analysis. A) **1a**, B) **1b**, C) **2**. His201 residue and ligands are depicted as sticks and portion of the S1′ loop backbone as cartoon.

The results emerging from Figures 8 and 9 might be better appreciated by examining in detail the conformations extracted from ED analysis performed on ligands using the same procedure already outlined for the enzyme structures, and reported in Figure 10. In particular, the compound **1a** in the enzyme assumes three conformations (Figure 10A). In the first and second conformations the ligand forms a π-π interaction with His201 by its pyridine, in the third by its furan ring. Changes of the ligand position and interactions do not induce a rearrangement of the S1′ loop. A similar behaviour has been observed in the four conformations assumed by the ligand **1b** (Figure 10B), except for the stable stacking interaction between His201 and pyridine.

The ligand **2** shows four conformations (Figure 10C), where it ensures always the π-π stacking with the furan and two H-bonds between the pyridyl amide CO and the Thr227 OH and between the pyridine N and the Thr229 OH. These interactions are maintained because the specificity loop changes its state adapting to the ligand conformations.

These data clearly indicate that the three ligands undergo a fluctuation decrease upon binding and their inclusion is accompanied by a quite different structural reorganization of the S1′ loop that results more stabilized with the inhibitors **1a** and **1b**, and moves more with ligand **2**.

In order to found all our hypotheses on more solid grounds, we have explicitly evaluated the differential binding free-energy and we also attempted to discriminate between enthaplic and entropic contribution.

#### Thermodynamic Integration

Any direct evaluation of binding free energy in large systems as the present one might be frustrated by its complexity. In fact, large amplitude motions revealed by previous analysis (Figure 6) clearly indicate that quantitative free energy evaluation using standard TI approaches, if not extended for prohibitively long simulation times, might be severely affected by the choice of the initial conditions. For this reason we decided to carry out TI integration starting from different initial enzyme configurations, selected from the previously described ED analysis. This, at least in principle, should reduce the systematic error due to the incompleteness of the phase-space sampling.

The extracted structures were selected within the spots obtained from projection of the trajectories onto the related C^α^ essential plane (Figure S2).

A first set of TI calculations were carried out at 300 K and a second set at 323 K in order to provide some information about the entropic and energetic factors affecting the ligand binding. In both sets we adopted, for each starting configuration, the computational scheme proposed by McCammon and coworkers [71].

Details of the TI trajectories are reported in the Supporting Information (Figures S3, S4, S5).

The results are collected in the Table 2 and indicate that at 300 K within the error, ligand **1b** shows the highest affinity toward MMP-2, although quite similar to **1a**. On the other hand ligand **2** shows the lowest affinity. These values are in line with those derived from inhibition data and calculated from the experimental IC_50_ [26].
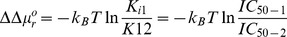
(1)this equivalence can be applied on the basis of the Cheng-Prousoff equation [72]:
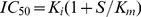
(2)that correlates the *IC_50_* to the *K_i_* for an enzyme inhibitor, knowing the substrate molar concentration (*S*) and the Michaelis constant (*Km*), which in the present case are equal for **1** and **2**.

**Table 2:** Delta Free-energy for binding reaction (ΔΔμ_r_°, kJ/mole).

Reaction	300 K	323 K
**1a**+MMP2 = MMP2:**1a**	16±12	11±16
**1b**+MMP2 = MMP2:**1b**	0	3±16
**2**+MMP2 = MMP2:**2**	79±26	75±26

According to the propagation of the standard errors from the TI integrations. Note that all data are reported taking the **1b** binding free energy at 300 K as reference.

As the IC_50_ for ligand **2** is not exactly specified, but is reported >17 µM (Figure 2) the *ΔΔμ_r_°* value, calculated on the basis of the equation (1) is ≥15 kJ/mole at 300 K, not in disagreement with our data.

A further important aspect concerns which of the two tautomers is actually more active. In principle this information might be derived by results in Table 2 from which it turns out that, although with a relatively high uncertainty, **1b** seems more active than **1a**.

**Figure 11:**
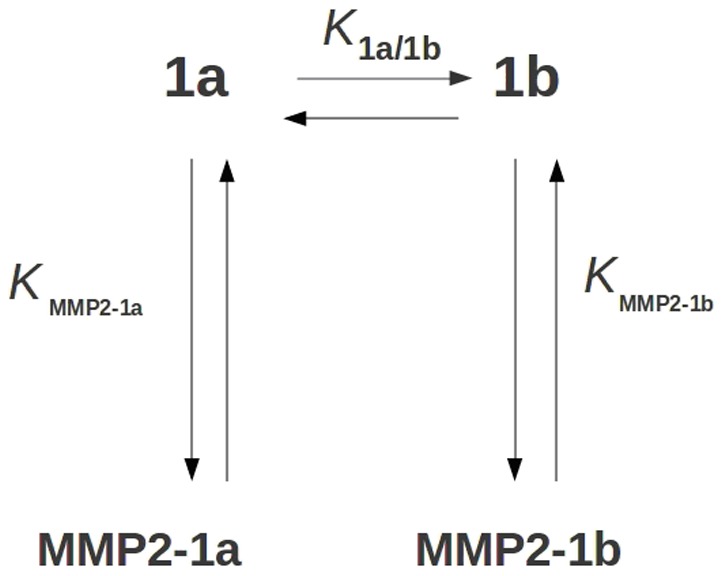
Representation of the binding process involving the tautomeric equilibrium between 1a and 1b.

However a complete and exhaustive picture can only be obtained after addressing the relative stability between **1b** and **1a** in aqueous solution. As a matter of fact if we consider the scheme reported in Figure 11, it turns out that the relative **1a**/**1b** affinity toward MMP-2, i.e. the ratio of [MMP-2:**1a**]/[MMP-2:**1b**] might be affected by the **1a**–**1b** interconversion and, hence, by their relative stability of the two isomers.

In fact using the scheme in Figure 11 the [MMP-2:**1a**]/[MMP-2:**1b**] quantity can be calculated using the equation:
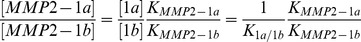
(3)where K_MMP2-1a_/K_MMP2-1b_ could be easily evaluated from the *ΔΔμ_r_°* reported in the Table 2 and 1/K_1a/1b_ has been calculated as described in the next session.

In order to address this further point we utilized QM calculations with details reported in the Materials and Methods and described in the next sub-section.

### QM calculations

Two main problems have to be faced when relative free-energies between molecular systems like **1a** and **1b** are concerned:

the effect of the solvent;
the presence of many almost degenerate conformations.

The presence of the solvent, as already pointed out in the Materials and methods section, has been taken into account using CPCM.

The presence of almost degenerate configurations was addressed by decreasing the complexity of the structures by considering only the moieties next to the pyrazole ring involved in the tautomerization.

Hence it is important to remark that we assume that the **1a/1b** equilibrium is not heavily affected by the presence of the moieties disregarded by our model.

**Figure 12:**
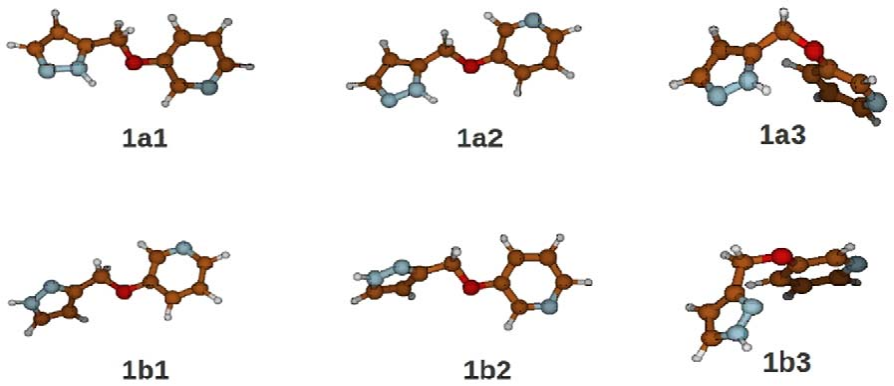
Schematic views of the species utilized for thermodynamical calculations (see Table 3).

The structures **1a1**, **1a2**, **1a3**, **1b1,1b2** and **1b3** schematically reported in Figure 12 were found as the most stable and almost degenerate both in gas-phase and in CPCM water solution (water dielectric) at the selected level of theory. The related energetic values are reported in Table 3. Details of the geometrical parameters are reported in the Supporting Information (Tables S1, S2, S3, S4, S5, S6).

**Table 3:** B3LYP/6-31+G* gas-phase absolute free-energies and solvation (excess) hydration free energies.

Species	Gas-phase relative molar free energies at 300 K (Δμ°_gas,species_, kJ/mol)	Solvation Free energy (Δμ°_hydration,species_, kJ/mol)
**1a1**	0	−72.0
**1a2**	−0.7	−67.8
**1a3**	3.2	−62.3
**1b1**	−3.8	−75.3
**1b2**	−3.6	−71.1
**1b3**	6.5	−76.6

For the gas-phase standard state, the solute at 1.0 M density and at 300 K was used. Standard statistical thermodynamics relations were utilized for evaluating Gibbs Free energies.

In order to study the equilibrium constant for the reaction
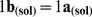
the following model, based on standard thermodynamic cycles, has been applied
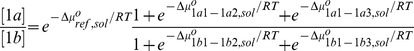
(4)where
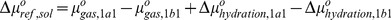
(5)and
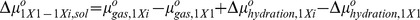
(6)with X being **a** or **b** and i = 2, 3

Using the data in Table 3 we obtained, for the equilibrium constant [**1a**]/[**1b**] at 300 K the value of 0.21 indicating that aqueous **1a**, including all the accessible conformers, shows a free energy about 4.2 kJ/mol higher than aqueous **1b**.

It means that **1b**, including all the accessible conformers, is thermodynamically more stable than **1a** and, hence more populated at room temperature in water solution. This result might be explained on the basis of the data reported in Table 3 from which emerges a higher gaseous basicity of N1 with respect to N2 and a larger hydration free energy of **1b** with respect to **1a**.

Introducing the value of 0.21 in equation (3), we obtain a value for the [MMP-2:**1a**]/[MMP-2:**1b**] ratio of 3.5*10^−4^.

This value clearly indicates that the species **1b** is the most stable and most active toward MMP-2.

## Conclusions

Our work presents a case study where more computational approaches have been applied to provide an explanation of the observed experimental activity of two ligands structurally related but with very different potency toward MMP-2 and representing an example of activity cliff.

This study confirms that for this target macromolecule, docking approach alone is not able to account for the complex consequences produced by the ligand binding because of the observed induced-fit and the dynamical-mechanical effects experienced by the system. Docking calculations, however, suggested for these molecules a binding mode not involving the zinc ion and confirmed by the MD analysis. Obviously, additional and more quantitative investigations would be necessary for excluding the possibility of the presence of zinc-binding configurations. Nevertheless the presently employed MD setup, along with the docking predicted binding mode, was validated by the reproduction of binding relative free energies in agreement with experimental data. In this respect species **1** (**1a** and **1b**) turns out to be more affine than **2** toward MMP-2. In addition our model suggests that **1b** is not only the most stable tautomer but also the most active ligand, even though this data are not supported, for the moment, by experimental observations.

In order to rationalize the above results, the role of enthalpic and entropic factors in the stabilization of the MMP-2 complexes was evaluated. Lack of a relevant temperature dependence of relative binding free energies (Table 2), allows us to consider that the main determinant for ligand affinity is not entropic but, rather, enthalpic.

In this respect, however, analysis of the binding mode does not immediately reveal drastic differences among the three species. As a matter of fact local interactions are not able to plausibly provide a direct and simple explanation to the greater affinity of **1** with respect to **2**, and in particular of **1b** with respect to **1a**. On the other hand other factors emerged from our study that probably play more important role. For example an increase of the number of intramolecular H-bonds formed between the S1′ site residues is found when ligand **1** binds to MMP-2. It probably means that the binding affinity of the active ligand might be related to its ability to produce significant structural stabilization, with respect to the free enzyme.

Our study indicates that the main difficulty associated to a full rationalization of a ligand affinity as well as to an effective structure-based design of new potential drugs, is related to the rather unpredictable mechanical-dynamical alterations of the receptor induced by the presence of the ligand. Moreover, the picture is even more complicated by the fact that small chemical differences of the ligand can produce, in some cases, dramatic modifications of the receptor conformational repertoire and, hence, drastic thermodynamical changes.

## Supporting Information

Figure S1 For better characterizing the conformational space spanned by the four investigated systems, i.e. the conformations sampled by MMP-2 in all investigated cases, the corresponding projection onto the plane characterized by the first two eigenvectors of the concatenated MMP-2 trajectories were extracted for the apo form (blue) and for the complexes with **1a** (black), **1b** (red) and **2** (green). When different concatenated trajectories produce perfectly superimposable projections it means that they span the same conformational space. On the other hand a scarce or partial overlap indicates differences in the sampled conformational space. The resulting 2D projections reported in Figure S1 basically show a partial overlap between the four trajectories. In particular the spots produced for the apo are never superimposed by the spots of the other three systems demonstrating that the presence of whatever ligand significantly modifies the conformational repertoire. For clarity sake, each trajectory projection is also shown separately. From the above spots we extracted the structures reported in the Figure 7 of the manuscript.
(TIF)

Figure S2 Representation of the extracted conformations (colored spots) from the ED analysis for each complex (A:**1a**, B:**1b**; C:**2**) utilized as starting conformations for TI calculations.
(TIF)

Figure S3 Curves for the Thermodynamic Integration for **1a** species.
(TIF)

Figure S4 Curves for the Thermodynamic Integration for **1b** species.
(TIF)

Figure S5 Curves for the Thermodynamic Integration for **2** species.
(TIF)

Table S1 Details of optimized Structure 1a1 from B3LYP/6-31+G(d) (Gaussian-like coordinates).
(DOC)

Table S2 Details of optimized Structure 1a2 from B3LYP/6-31+G(d) (Gaussian-like coordinates).
(DOC)

Table S3 Details of optimized Structure 1a3 from B3LYP/6-31+G(d) (Gaussian-like coordinates).
(DOC)

Table S4 Details of optimized Structure 1b1 from B3LYP/6-31+G(d) (Gaussian-like coordinates).
(DOC)

Table S5 Details of optimized Structure 1b2 from B3LYP/6-31+G(d) (Gaussian-like coordinates).
(DOC)

Table S6 Details of optimized Structure 1b3 from B3LYP/6-31+G(d) (Gaussian-like coordinates).
(DOC)
