# Supplementary figures and images for: An Integrated Computational Approach to Rationalize the Activity of Non-Zinc-Binding MMP-2 Inhibitors
Source: PLoS One. 2012 Nov 8;7(11):e47774. doi: 10.1371/journal.pone.0047774 (PMC3493580; doi:10.1371/journal.pone.0047774)

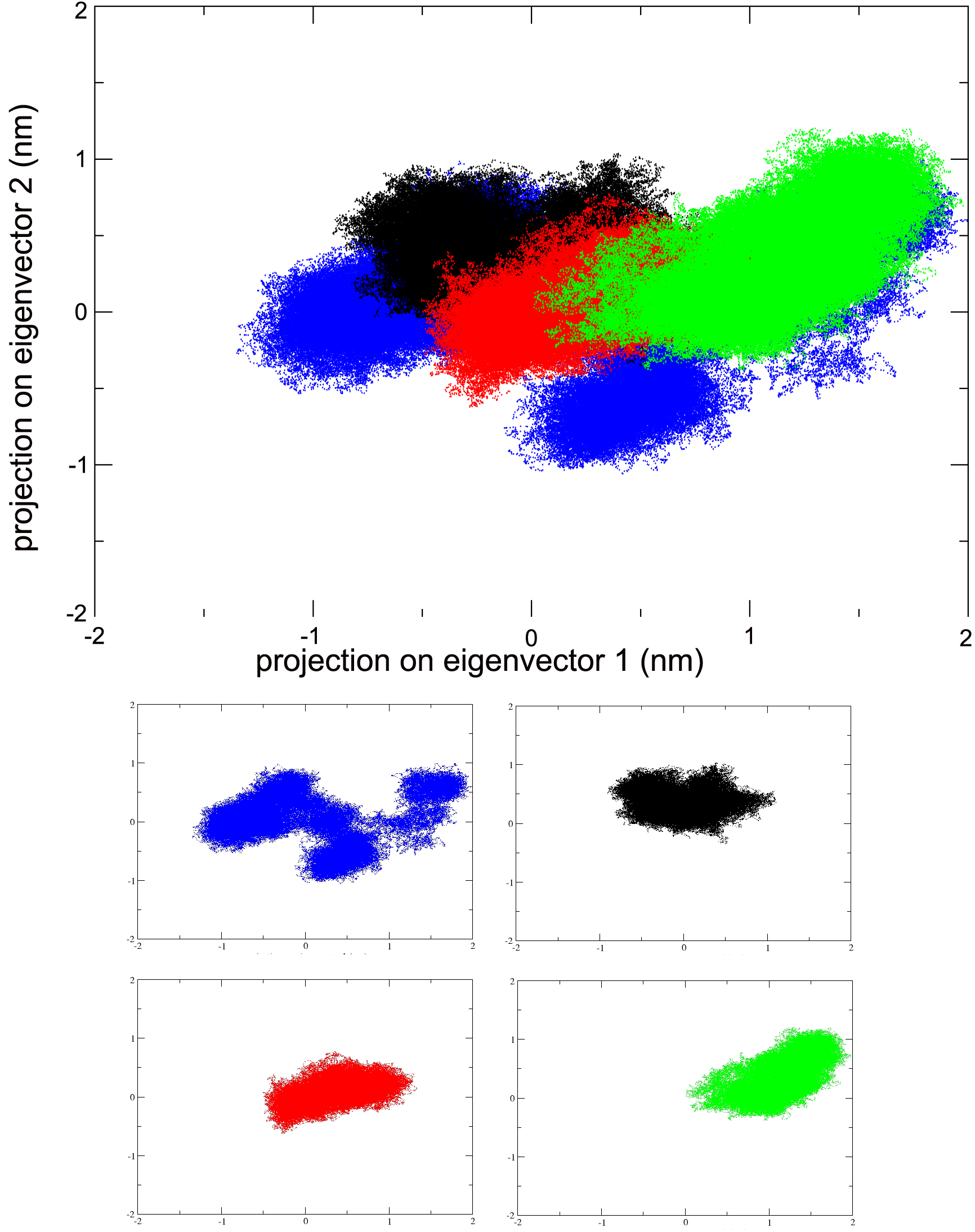

Supplement: Figure S1 — For better characterizing the conformational space spanned by the four investigated systems, i.e. the conformations sampled by MMP-2 in all investigated cases, the corresponding projection onto the plane characterized by the first two eigenvectors of the concatenated MMP-2 trajectories were extracted for the apo form (blue) and for the complexes with 1a (black), 1b (red) and 2 (green). When different concatenated trajectories produce perfectly superimposable projections it means that they span the same conformational space. On the other hand a scarce or partial overlap indicates differences in the sampled conformational space. The resulting 2D projections reported in Figure S1 basically show a partial overlap between the four trajectories. In particular the spots produced for the apo are never superimposed by the spots of the other three systems demonstrating that the presence of whatever ligand significantly modifies the conformational repertoire. For clarity sake, each trajectory projection is also shown separately. From the above spots we extracted the structures reported in the Figure 7 of the manuscript. (TIF) [file pone.0047774.s001.tif]

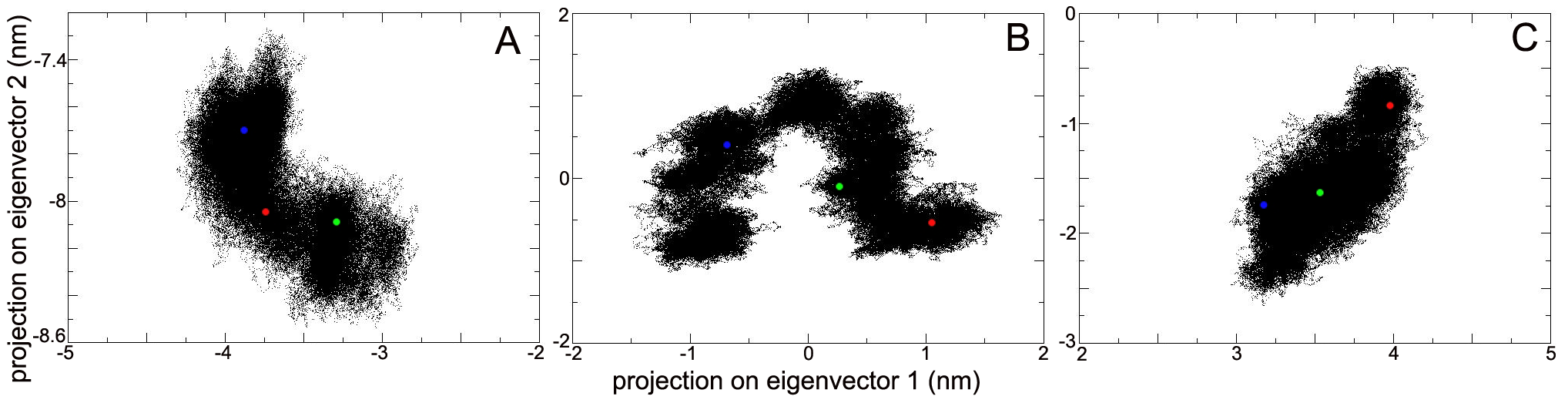

Supplement: Figure S2 — Representation of the extracted conformations (colored spots) from the ED analysis for each complex (A:1a, B:1b; C:2) utilized as starting conformations for TI calculations. (TIF) [file pone.0047774.s002.tif]

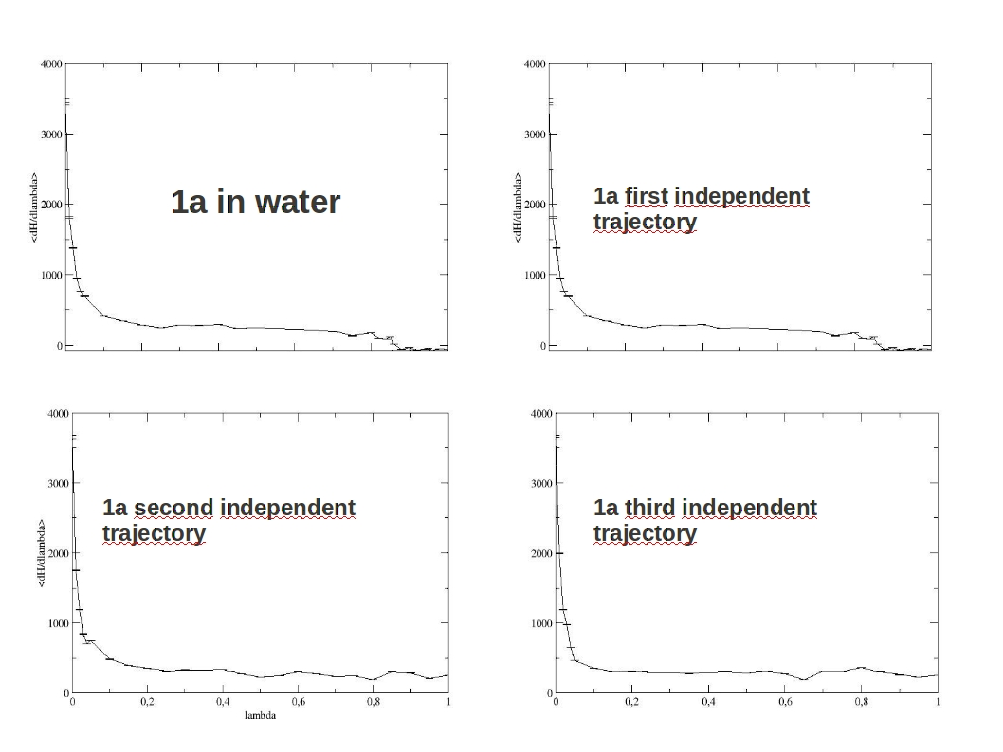

Supplement: Figure S3 — Curves for the Thermodynamic Integration for 1a species. (TIF) [file pone.0047774.s003.tif]

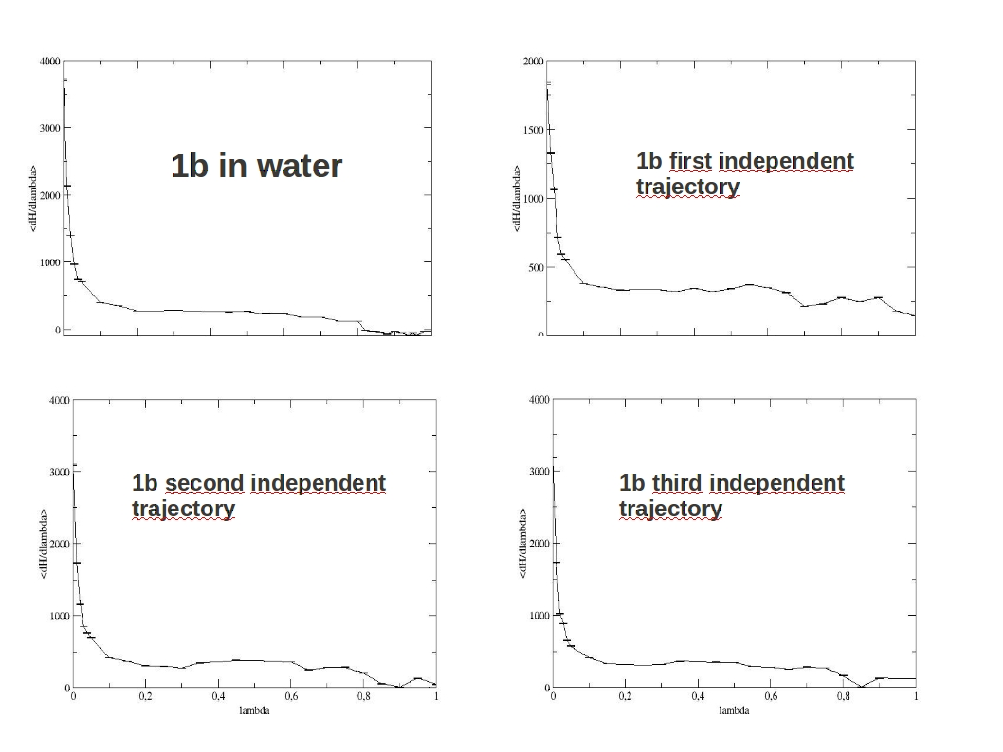

Supplement: Figure S4 — Curves for the Thermodynamic Integration for 1b species. (TIF) [file pone.0047774.s004.tif]

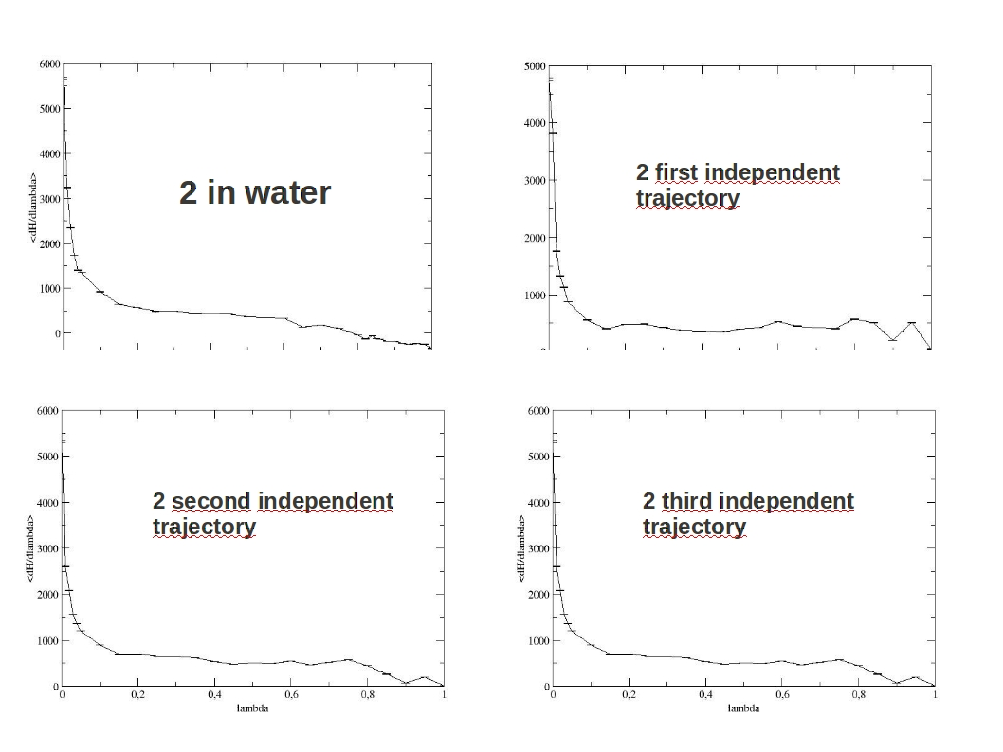

Supplement: Figure S5 — Curves for the Thermodynamic Integration for 2 species. (TIF) [file pone.0047774.s005.tif]
