# Supplementary material for: An Integrated Computational Approach to Rationalize the Activity of Non-Zinc-Binding MMP-2 Inhibitors
Source: PLoS One. 2012 Nov 8;7(11):e47774. doi: 10.1371/journal.pone.0047774 (PMC3493580; doi:10.1371/journal.pone.0047774)
Supplement: Table S1 — Details of optimized Structure 1a1 from B3LYP/6-31+G(d) (Gaussian-like coordinates). (DOC) [file pone.0047774.s006.doc]

**Table S1. Details of optimized Structure 1a1 from B3LYP/6-31+G(d) (Gaussian-like coordinates)**

---------------------------------------------------------------------

Center Atomic Atomic Coordinates (Angstroms)

Number Number Type X Y Z

---------------------------------------------------------------------

1 7 0 3.592308 -1.233473 -0.260169

2 6 0 2.261951 -1.241807 -0.306072

3 6 0 1.472519 -0.100269 -0.077950

4 6 0 2.113020 1.106889 0.210289

5 6 0 3.511445 1.111381 0.258209

6 6 0 4.206546 -0.071701 0.018904

7 8 0 0.119379 -0.281361 -0.166941

8 6 0 -0.735337 0.832558 0.115631

9 6 0 -2.151908 0.373878 0.005408

10 6 0 -3.281234 0.994963 -0.503087

11 6 0 -4.335078 0.083621 -0.255017

12 7 0 -3.901641 -1.022481 0.349724

13 7 0 -2.577249 -0.814900 0.512298

14 1 0 -5.384351 0.181739 -0.500508

15 1 0 -3.334493 1.963821 -0.980198

16 1 0 1.772881 -2.186447 -0.535979

17 1 0 1.558485 2.021196 0.392003

18 1 0 4.048630 2.029677 0.479041

19 1 0 5.293247 -0.097643 0.049719

20 1 0 -0.514955 1.204475 1.127692

21 1 0 -0.557316 1.648252 -0.596649

22 1 0 -2.002629 -1.555291 0.889544
