# Supplementary material for: An Integrated Computational Approach to Rationalize the Activity of Non-Zinc-Binding MMP-2 Inhibitors
Source: PLoS One. 2012 Nov 8;7(11):e47774. doi: 10.1371/journal.pone.0047774 (PMC3493580; doi:10.1371/journal.pone.0047774)
Supplement: Table S2 — Details of optimized Structure 1a2 from B3LYP/6-31+G(d) (Gaussian-like coordinates). (DOC) [file pone.0047774.s007.doc]

**Table S2. Details of optimized Structure 1a2 from B3LYP/6-31+G(d) (Gaussian-like coordinates)**

---------------------------------------------------------------------

Center Atomic Atomic Coordinates (Angstroms)

Number Number Type X Y Z

---------------------------------------------------------------------

1 7 0 2.597109 0.796007 0.552485

2 6 0 2.148094 -0.366678 0.005931

3 6 0 3.259833 -0.976030 -0.552601

4 6 0 4.327959 -0.085110 -0.291663

5 7 0 3.919261 0.998110 0.369288

6 6 0 0.728990 -0.813970 0.125564

7 8 0 -0.118280 0.310115 -0.149353

8 6 0 -1.473990 0.137284 -0.075416

9 6 0 -2.262499 1.266784 -0.328742

10 6 0 -3.644831 1.139177 -0.266311

11 6 0 -4.195811 -0.109871 0.046474

12 7 0 -3.450622 -1.191584 0.286982

13 6 0 -2.115928 -1.072024 0.226649

14 1 0 5.370816 -0.183448 -0.562979

15 1 0 3.292282 -1.923906 -1.071789

16 1 0 -1.780963 2.210236 -0.569582

17 1 0 -1.553455 -1.979350 0.426668

18 1 0 -5.273629 -0.244977 0.104227

19 1 0 0.512049 -1.186431 1.137702

20 1 0 0.538287 -1.625530 -0.587374

21 1 0 2.039448 1.524888 0.974650

22 1 0 -4.289725 1.992496 -0.457295
