# Supplementary material for: An Integrated Computational Approach to Rationalize the Activity of Non-Zinc-Binding MMP-2 Inhibitors
Source: PLoS One. 2012 Nov 8;7(11):e47774. doi: 10.1371/journal.pone.0047774 (PMC3493580; doi:10.1371/journal.pone.0047774)
Supplement: Table S6 — Details of optimized Structure 1b3 from B3LYP/6-31+G(d) (Gaussian-like coordinates). (DOC) [file pone.0047774.s011.doc]

**Table S6. Details of optimized Structure 1b3 from B3LYP/6-31+G(d) (Gaussian-like coordinates)**

---------------------------------------------------------------------

Center Atomic Atomic Coordinates (Angstroms)

Number Number Type X Y Z

---------------------------------------------------------------------

1 6 0 2.435123 -0.999506 -0.509486

2 6 0 1.276273 -0.565272 0.156740

3 6 0 1.224841 0.750389 0.620665

4 6 0 2.341433 1.563798 0.401803

5 6 0 3.446867 1.042712 -0.267552

6 7 0 3.495299 -0.220815 -0.719799

7 1 0 2.483294 -2.023471 -0.875059

8 1 0 0.346570 1.147177 1.117343

9 1 0 2.342049 2.595129 0.743714

10 1 0 4.327216 1.653732 -0.452818

11 8 0 0.281422 -1.501741 0.285642

12 6 0 -0.864075 -1.208649 1.093611

13 6 0 -1.863868 -0.331436 0.398967

14 1 0 -1.290317 -2.194574 1.305970

15 1 0 -0.564744 -0.759596 2.048696

16 6 0 -2.723387 0.649759 0.868721

17 6 0 -3.460712 1.057797 -0.268728

18 7 0 -3.089748 0.394320 -1.363542

19 7 0 -2.130326 -0.451034 -0.929877

20 1 0 -1.652422 -1.041905 -1.596213

21 1 0 -4.236768 1.808881 -0.336444

22 1 0 -2.807789 1.013699 1.883744
